# Fabrication and Characterization of Hybrid Films Based on NiFe_2_O_4_ Nanoparticles in a Polymeric Matrix for Applications in Organic Electronics

**DOI:** 10.3390/nano13091525

**Published:** 2023-04-30

**Authors:** María Elena Sánchez Vergara, María José Agraz Rentería, América R. Vázquez-Olmos, Karen L. Rincón-Granados, José Ramón Álvarez Bada, Roberto Y. Sato-Berrú

**Affiliations:** 1 Faculty of Engineering, Universidad Anáhuac México, Avenida Universidad Anáhuac 46, Col. Lomas Anáhuac, Huixquilucan 52786, Estado de México, Mexico; maria.agrazre@anahuac.mx (M.J.A.R.); ramon.alvarez@anahuac.mx (J.R.Á.B.); 2 Institute of Applied Sciences and Technology, Universidad Nacional Autónoma de México, Circuito Exterior S/N, C.U., Coyoacán 04510, Ciudad de México, Mexico; karenloraine08@gmail.com (K.L.R.-G.); roberto.sato@icat.unam.mx (R.Y.S.-B.)

**Keywords:** spinel, NiFe_2_O_4_ NPs, hybrid film, optical properties, electronic device

## Abstract

Hybrid films for applications in organic electronics from NiFe_2_O_4_ nanoparticles (NPs) in poly(3,4 ethylene dioxythiophene), poly(4-styrenesulfonate) (PEDOT:PSS), and poly(methyl methacrylate) (PMMA) were fabricated by the spin-coating technique. The films were characterized by infrared spectroscopy, atomic force microscopy, scanning electron microscopy, and energy-dispersive spectroscopy to subsequently determine their optical parameters. The electronic transport of the hybrid films was determined in bulk heterojunction devices. The presence of NiFe_2_O_4_ NPs reinforces mechanical properties and increases transmittance in the hybrid films; the PEDOT:PSS-NiFe_2_O_4_ NPs film is the one that has a maximum stress of 28 MPa and a Knoop hardness of 0.103, while the PMMA-NiFe_2_O_4_ NPs film has the highest transmittance of (87%). The Tauc band gap is in the range of 3.78–3.9 eV, and the Urbach energy is in the range of 0.24–0.33 eV. Regarding electrical behavior, the main effect is exerted by the matrix, although the current carried is of the same order of magnitude for the two devices: glass/ITO/polymer-NiFe_2_O_4_ NPs/Ag. NiFe_2_O_4_ NPs enhance the mechanical, optical, and electrical behavior of the hybrid films and can be used as semi-transparent anodes and as active layers.

## 1. Introduction

Spinel ferrites with general formula AB_2_O_4_ (where A is a divalent cation and B is Fe^3+^), are amongst the most studied iron-based systems. As nanomaterials, spinel ferrites are interesting due to their magnetic properties, which suggests their potential applications, as follows: in ferrofluidics [1] and high-density memory systems [2]; as adsorbents and catalysts for water decontamination and treatment [3,4]; as contrast agents in magnetic resonance imaging [5]; and in controlled drug delivery [6,7], hyperthermia [8,9,10,11], and other biomedical applications. Moreover, ferrites have applications in high-frequency drive technologies due to their low conductivity as well as their low current loss. Ferrites are high-resistivity dielectrics; therefore, they are used in the fabrication of transformer cores operating at high frequencies, in audiovisual memory storage, and in recording heads [12]. Depending on the cations making up a ferrite, it will have different thermal, electrical, magnetic, optical, and mechanical (etc.) properties [13]. In particular, nickel ferrite (NiFe_2_O_4_) is an inverse spinel-type oxide with a bandgap of 1.5–5.0 eV for the bulk material. This ferrite crystallizes in a cubic system with space group Fd-3m and has low coercivity and saturation magnetization values as well as a high electrical resistivity; these properties make it a suitable material for magnetic and magneto-optical applications [14]. Moreover, NiFe_2_O_4_ nanoparticles have a near-zero hysteresis loss, which makes them a suitable core material for power transformers as well as in telecommunication and electronics applications [14,15,16]. They also have catalytic, electrical and electronics applications as a consequence of their gas- and humidity-sensing properties [14,17]. The preparation conditions and the techniques applied to the production of NiFe_2_O_4_ NPs strongly influence their properties [18]; the shape of the particles, grain size, and distribution (etc.) are also important factors [14]. Brook and Kingery [19] reported that in NiFe_2_O_4_ samples, ferrimagnetism is associated with polycrystalline spinels with a grain size greater than or equal to 15 nm, while superparamagnetism is associated with spinels of smaller grain size, around 10 nm, and paramagnetism is found in noncrystalline samples [14]. On the other hand, Nathani and Misra [20] reported that interparticle interactions strongly affect the magnetic properties of NiFe_2_O_4_ nanoparticles [14].

Although there is a considerable number of studies on magnetic properties and their relationship with the structure and morphology of NiFe_2_O_4_ NPs, there is little information about other types of properties, such as optical and electrical types, that may facilitate the use of NiFe_2_O_4_ NPs in electronic organic applications. Unfortunately, NPs tend to agglomerate and they can oxidize in the presence of air [21]. Moreover, their intrinsic electrical conductivity is low [22] and they have poor mechanical properties such as low tensile strength and Young’s modulus, which limits their use in electronic devices. Thus, the study of the optical and electrical properties of NiFe_2_O_4_ NPs should be carried out in films or layers, thus allowing the nanoparticles to be deposited as active or electric-charge transporter layers for electronic devices. In the fabrication of films and layers, NPs are mainly coated with conductive polymers which have been widely used in photolytic devices and supercapacitors [21,23]. Conductive polymers exhibit substantial conductivity in the doping state, as well as lower band gaps, when compared to conventional polymers [23]. Bearing this in mind, the novelty of this work is related to the fabrication of hybrid films formed by NiFe_2_O_4_ NPs embedded in polymer matrices of poly(3,4-ethylenedioxythiophene)-poly(styrenesulfonate) (PEDOT:PSS) and poly(methacrylic acid methyl ester) (PMMA), which allows for applications in organic electronics.

PEDOT:PSS is one of the most popular polymers used in organic electronics because of its characteristics of high stability, excellent conductivity, transparency, flexible mechanical properties, and its capacity to act as a hole transport layer [24]. PEDOT exhibits stability and a high conductivity at an oxidized state, while PSS acts as a counter-ion to ensure the electroneutrality of PEDOT:PSS and facilitates the dispersion of the polymer in an aqueous medium for film deposition. On the other hand, PMMA has been highlighted for use in electronics devices and optical components, such as polymeric waveguides, because of its transparency and flexibility [25], its volume productivity, and its low cost [26]. Additionally, PMMA in film devices permits a stable electronic performance [25,27] and acts as a sacrificial layer to enable free-standing electronics [25,28,29]. The combination of PEDOT:PSS and PMMA with chemically different structures, such as NiFe_2_O_4_ NPs, favors the chemical stability of NPs under service conditions [23] and may decrease the energy band gap and charge-transfer resistance, which improves the optical and electrical properties of composite films and facilitates their use in various electronic applications [23,30,31,32].

## 2. Materials and Methods

### 2.1. NiFe_2_O_4_NPs Characterization

All the chemical reagents, metallic salts, solvents, and polymers used in this work were purchased from commercial sources (Sigma-Aldrich, St. Louis, MO, USA). The NiFe_2_O_4_ NPs were obtained according to procedures previously reported by Rincon-Granados et al. from nickel acetylacetonate (Ni(C_5_H_7_O_2_)_2_) (95%), iron acetylacetonate (Fe(C_5_H_7_O_2_)_3_) (99.9%), and sodium hydroxide (NaOH) (97%) by a mechanochemical procedure [33]. X-ray diffraction patterns were performed at room temperature with Cu K *α* radiation (*λ* = 1.5406 Å) in a D5000 Siemens diffractometer (Bruker, Billerica, MA, USA); diffraction intensity was measured between 10° and 70° with a 2*θ* step of 0.01° and 0.25 s per point. The average crystal size (D) of the NPs was estimated from their diffractograms using the Debye-Scherer formula, *D* = *κλ*/*β*cos *θ*, where *κ* is the shape factor equal to 0.9, *λ* is the CuK *α* radiation, *β* is the full width at half maximum intensity (FWHM) of the selected peaks, and *θ* is the Bragg angle. Transmission electron micrographs (TEM) were obtained with an FEI Tecnai F20 microscope (Thermo Fisher, Waltham, MA, USA) operating at 200 kV S/TEM field emission with an X-TWIN lens and a high-brightness field emission electron gun (FEG).

### 2.2. Hybrid Film Deposition

Hybrid films composed of NiFe_2_O_4_ NPs embedded in poly(3,4-ethylenedioxythiophene):poly(styrenesulfonate) (PEDOT:PSS: [C_8_H_8_O_3_S]_n_-[C_6_H_6_O_2_S]_n_) and poly(methacrylic acid methyl ester) (PMMA; [CH_2_C(CH_3_)(CO_2_CH_3_)]_n_) matrices were fabricated using the spin-coating technique. PEDOT:PSS and NiFe_2_O_4_ NPs, as well as PMMA and NiFe_2_O_4_ NPs, were dispersed using the G560 shaker of Scientific Industries Vortex-Genie (Bohemia, New York, USA). Dispersions at 5 wt.% of NPs were used in (i) 1 wt.% PEDOT:PSS in water or (ii) 1 wt.% PMMA in chloroform. The dispersions were deposited with a Smart Coater 200 equipment (Laurell Technologies Corporation, North Wales, PA, USA) that operated, for the PEDOT: PSS-NiFe_2_O_4_ NPs films, at a constant angular speed of 800 rpm with a time of 8 s and an acceleration of 250 rpm/s. The constant angular speed used for the PMMA-NiFe_2_O_4_ NPs films was 600 rpm with a time of 9 s and an acceleration of 250 rpm/s. Different substrates were used to support the films: Corning glass, monocrystalline silicon wafers (c-Si), and glass coated with indium tin oxide (ITO). Initially, the Corning glass and the ITO glass substrates were sequentially washed in an ultrasonic bath with dichloromethane, methanol, and acetone. The silicon substrates were washed with a “p” solution (10 mL HF, 15 mL HNO_3_, and 300 mL H_2_O) to remove surface oxide. After deposition, the films were dried at 85 °C for 2 min on a hot plate. The PEDOT:PSS-NiFe_2_O_4_ NPs films underwent a post-treatment by vapor exposure of isopropanol (IPA) and were heated at 40 °C for 10 min.

### 2.3. Hybrid Film Characterization

FTIR spectroscopy analysis was performed using a Nicolet iS5-FT spectrometer (Thermo Fisher Scientific Inc., Waltham, MA, USA) at a wavelength range of 4000 to 400 cm^−1^. Raman spectra of 100 to 900 cm^−1^ were acquired in a Nicolet Almega XR (Thermo Scientific Nicolet, Waltham, MA, USA) dispersive Raman spectrometer and detected by a CCD camera at 25 s and a resolution of ~4 cm^−1^. The excitation beam was a Nd:YVO 4532 nm laser, and the incident power on the sample was ~3 mW. The roughness, topography, thickness, and mechanical properties of the hybrid films deposited on the silicon substrate were investigated with an atomic force microscope (AFM) using an Ntegra platform (Nanosurf, Liestal, Switzerland). In order to measure film thickness, an edge of the film was initially removed from the substrate in order to have a clean area so that only the substrate would be present and thus generate a step between the film and the substrate. The AFM tip was then placed on the film surface and made to pass through it in contact mode until it touched the clean substrate [34,35]. Subsequently, the images were analyzed with the Gwyddion software platform to determine the thickness through the height difference between the highest point and the lowest point. Due to the structural difference of the films and the type of technique used for the deposit, their average thicknesses changed slightly: 7.6 μm (PEDOT:PSS), 11.1 μm (PEDOT:PSS-NiFe_2_O_4_ NPs), 6.5 μm (PMMA) and 11.7 μm (PMMA-NiFe_2_O_4_ NPs).

To study the morphology and ensure the presence of the NiFe_2_O_4_ NPs in the films deposited on the glass substrate, energy-dispersive spectroscopy (EDS) and scanning electron microscopy (SEM) were performed on a ZEISS EVO LS 10 scanning electron microscope (Carl Zeiss AG, Oberkochen, Germany) with a coupled Bruker microanalysis system (Bruker Nano GmbH, Berlin, Germany). Optical properties, absorbance, and transmittance of the hybrid films on the glass substrate were obtained using a UV–Vis 300 Unicam spectrophotometer (Thermo Fisher Scientific Inc., Waltham, MA, USA), in a wavelength range from 190 to 1100 nm. An auto-ranging picoammeter Keithley 4200-SCS-PK1 (Tektronix Inc., Beaverton, OR, USA) with a four-point probe and a lighting controller circuit from Next Robotix (Comercializadora KMox, S.A. de C.V., Mexico City, Mexico), was used to obtain the electrical properties through current–voltage (I–V) measurements of the fabricated devices, using an ITO glass substrate as the anode and silver as the cathode. Finally, to modify the behavior of the device, a 2,3,7,8,12,13,17,18-octaethyl-21H,23H-porphine iron(III) chloride (PFeCl: C_36_H_44_ClFeN_4_) film was deposited over PEDOT:PSS-NiFe_2_O_4_ NPs and PMMA-NiFe_2_O_4_ NPs films using a high-vacuum thermal evaporation system (Intercovamex, Morelos, Mexico). The PFeCl film was heated to 250 °C and sublimated at a vacuum pressure of 2.6 × 10^−6^ Torr; the deposition rate was 35.1 Å/s.

## 3. Results and Discussion

**Figure 1:**
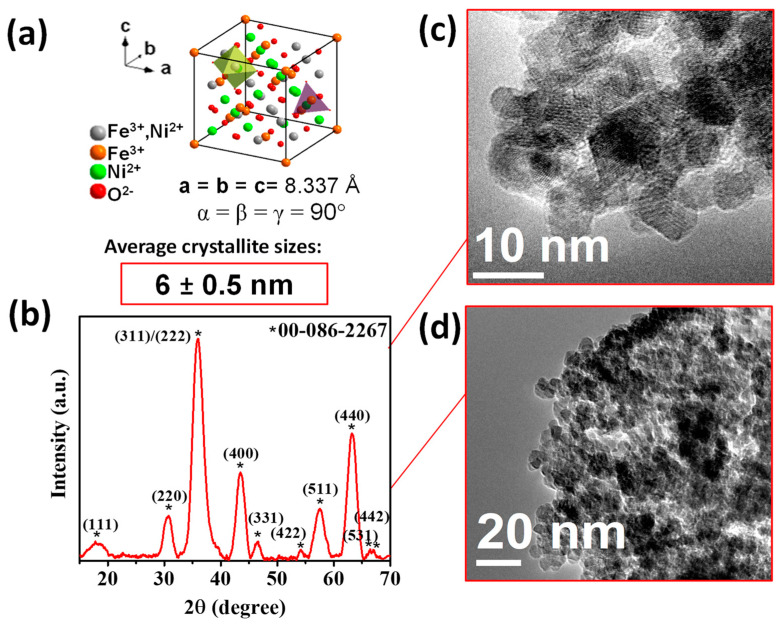
(**a**) Scheme of the crystalline structure of NiFe2O4, (**b**) X-ray diffraction pattern of the NiFe_2_O_4_ nanopowder, the asterisks correspond to the crystallographic planes reported on card 00-086-226, and (**c**,**d**) its corresponding TEM micrographs.

The NiFe_2_O_4_ NPs were characterized by powder XRD and TEM microscopy. Figure 1 shows the XRD pattern of the NiFe_2_O_4_ NPs (Figure 1a), which corresponds to an inverse spinel-type structure and a cubic phase with space group Fd-3 m and crystal lattice parameter a = 8.33 Å according to the ICDD 00-086-2267 card. No additional peaks from other faces were observed (Figure 1b). Scherrer’s equation estimated the average crystallite size at 6 ± 0.5 nm. TEM micrographs (Figure 1c,d) also corroborated the formation of nanocrystals with dimensions close to those determined from the corresponding X-ray diffraction pattern.

**Figure 2:**
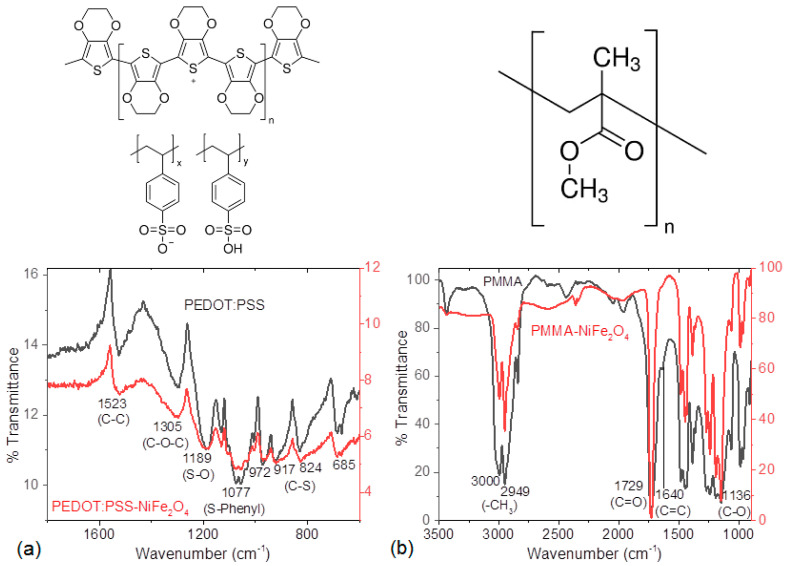
Structure and IR spectra of (**a**) PEDOT:PSS and (**b**) PMMA films.

Hybrid films were fabricated through the spin-coating technique. After deposition, IR spectroscopy measurements of the PEDOT:PSS-NiFe_2_O_4_ NPs and PMMA-NiFe_2_O_4_ NPs films were performed in order to verify that no degradation of the polymer matrix had taken place during deposition. In Figure 2a, the spectrum of the PEDOT:PSS-NiFe_2_O_4_ NPs film shows peaks corresponding to the polymer at (i) 1523 cm^−1^ for the C-C bond, (ii) 1305 cm^−1^ for the C-O-C bond, (iii) 1077 cm^−1^ for the S-O bond, and (iv) 1077 cm^−1^ for the bond between S and the phenyl group in PEDOT:PSS; (v) the last four peaks at 972, 917, 824, and 685 cm^−1^ reflect C-S group stretches in PEDOT:PSS [36,37,38]. Regarding PMMA, Figure 2b shows the IR spectrum for the PMMA-NiFe_2_O_4_ NPs film. The following signals corresponding to the polymer can be observed: (i) 3000 and 2949 cm^−1^ for the methyl group, (ii) 1729 cm^−1^ for the C=O bond, (iii) 1640 cm^−1^ for the C=C bond, and (iv) 1136 cm^−1^ for the C-O bond [39]. IR spectroscopy results indicate that the polymers used as a matrix in the fabrication of the hybrid films did not undergo decomposition during the deposition process. Regarding the NPs, due to their chemical nature and resistance to organic solvents and their resistance to high temperatures, no decomposition was expected during the preparation of the hybrid films. However, their distribution in the polymeric matrix was verified later in the morphological studies of the hybrid films.

**Figure 3:**
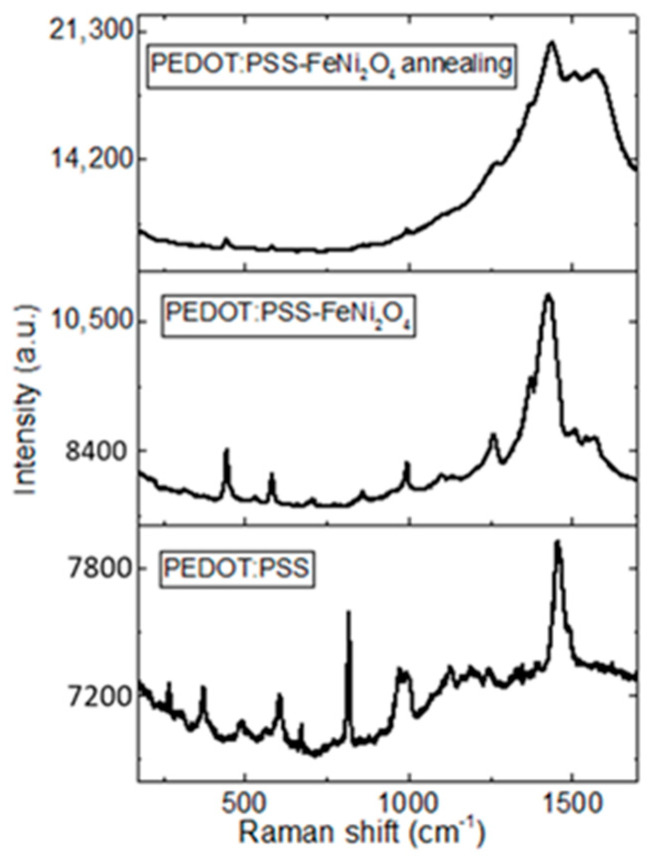
Raman spectra of the PEDOT:PSS film and the PEDOT:PSS-NiFe_2_O_4_ NPs film before and after the IPA post-treatment.

Raman spectroscopy was used to monitor the distribution of NPs in the polymeric matrix and the possible transformation of the benzoid structure to the quinoid structure of PEDOT before and after its treatment with IPA. Figure 3 shows the Raman spectra of PEDOT:PSS pristine and PEDOT:PSS-NiFe_2_O_4_ NPs films. The post-treated PEDOT:PSS-NiFe_2_O_4_ NPs film had a change in relative intensity in comparison to the PEDOT:PSS pristine film. Changes in the Raman spectral intensity confirm the interaction between the NiFe_2_O_4_ NPs and PEDOT chains; this may be caused by the redistribution of PEDOT:PSS and the NPs as a result of the IPA treatment. The increase in relative intensity for the treated film means that the PEDOT:PSS distribution becomes more uniform through the film, enhancing the detection of their main planes in the Raman spectrum. Additionally, the strongest band between 1380 and 1620 cm^−1^ corresponds to the C_α_ = C_β_ symmetric stretching of the five-membered thiophene ring on the PEDOT chains [40,41,42]. The symmetric stretching vibration C_α_ = C_β_ has a red-shifted peak when the chain structure changes from the benzoid to the quinoid form [36,37,38]. The displacement of the 1430 cm^−1^ signal in the spectra in Figure 3 is practically negligible, which is an indication that in this film the transformation of the PEDOT structure was not carried out. This result is relevant because the presence of NPs inhibits the transformation of PEDOT to the quinoid form; however, the post-treatment with IPA helps to improve the distribution of NPs in the polymeric matrix.

**Figure 4:**
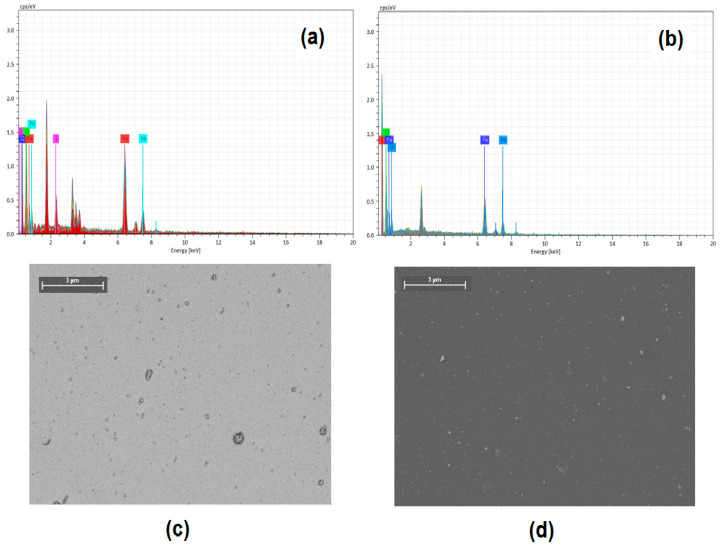
EDS microanalysis of (**a**) PEDOT:PSS-NiFe_2_O_4_ NPs and (**b**) PMMA-NiFe_2_O_4_ NPs films. SEM photomicrographs at 250× of (**c**) PEDOT:PSS-NiFe_2_O_4_ NPs and (**d**) PMMA-NiFe_2_O_4_ NPs films.

In the EDS analysis of the films after the IPA treatment, as shown in Figure 4a,b, the chemical elements corresponding to the polymer and the NPs can be observed. In both spectra, the presence of Ni and Fe is due to the NPs, and the presence of S and C in Figure 4a is due to the presence of PEDOT:PSS. The presence of O is due to the polymer as well as the NiFe_2_O_4_ NPs. As for the PMMA films, the presence of C in Figure 4b is due to the polymer and that of O is related to the NPs. SEM analysis (Figure 4c,d) was used to study the morphology of the films; a homogeneous distribution of NPs throughout the entire matrix was observed in PEDOT:PSS-NiFe_2_O_4_ NPs and PMMA-NiFe_2_O_4_ NPs. However, in the PEDOT:PSS film some NP agglomerations were observed which produced larger structures than those observed in the PMMA film. According to the EDS and SEM results, it is evident that the NiFe_2_O_4_ NPs were adequately incorporated into the polymer matrix, thus forming dispersed heterojunction films; however, a further topographic study of the films was necessary due to the important requirements in organic electronics related to the homogeneity and quality of the films needed in these devices.

**Figure 5:**
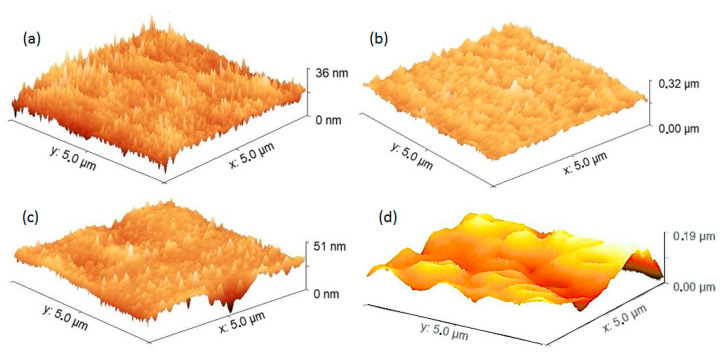
AFM images of (**a**) PEDOT:PSS, (**b**) PEDOT:PSS-NiFe_2_O_4_ NPs, (**c**) PMMA, and (**d**) PMMA-NiFe_2_O_4_ NPs films.

The topographic characterization of the hybrid films was supplemented by means of AFM studies. Figure 5 shows the AFM images for the films of the pristine PEDOT:PSS and PMMA polymers (Figure 5a,c, respectively) and for the hybrid PEDOT:PSS-NiFe_2_O_4_ NPs and PMMA-NiFe_2_O_4_ NPs films (Figure 5b,d, respectively). In the case of the films with PEDOT:PSS, a different topography is observed in terms of the growth direction of the polymer; in the pristine film, the direction is perpendicular to the surface (~90°), and in the PEDOT:PSS-NiFe_2_O_4_ NPs film with IPA post-treatment, a preferential growth direction is observed with an angle smaller than 90°. The interaction between NiFe_2_O_4_ and PEDOT:PSS can be a decisive factor in the topography of the film because NPs can behave as nuclei around which the polymer is deposited in a preferential direction. In Table 1, the root mean square roughness (RMS) and the average roughness (Ra) are presented; RMS represents the average of the squared deviations concerning the average height of the films and is more significant than Ra, which represents the arithmetic average of the absolute values of the heights of the films. The PEDOT:PSS pristine film had a lower roughness than the PEDOT:PSS-NiFe_2_O_4_ NPs film. This result is to be expected considering that the NPs have an average crystallite size of 5 ± 0.4 nm [33] and that when they are embedded in the PEDOT:PSS matrix they increase the roughness of the film. In the case of the PMMA polymer films, the topography, RMS, and Ra changed drastically upon incorporating NiFe_2_O_4_ NPs. The presence of NPs not only increases roughness but also allows the topography to become heterogeneous, with a larger grain size. When comparing the PEDOT:PSS films with the PMMA films, it is observed that although RMS and Ra do not differ significantly, their topographies are very different from those of the PEDOT:PSS-NiFe_2_O_4_ NPs and PMMA-NiFe_2_O_4_ NPs films. PEDOT:PSS seems to have better interaction and behavior as a matrix and generates more homogeneous films than PMMA.

Regarding mechanical behavior when considering a maximum applied force of 900 N, the maximum stress (σ_max_), the unitary deformation (ε_unit_), and the Knoop micro hardness (HK) were obtained by AFM and are presented in Table 1. In the case of films with PEDOT:PSS, it is observed that the presence of NiFe_2_O_4_ NPs increases the stress that the film is capable of withstanding by a factor of about 2.6 times. In the case of PMMA films, there is a very small increase in the stress that the film can withstand when introducing NiFe_2_O_4_ NPs. The above may be a result of the increase in grain size observed in the PMMA-NiFe_2_O_4_ NPs film (Figure 5d) and a deficiency of this polymer in its function as a matrix. On the other hand, HK was calculated from the length of the long penetration diagonal in the films, and according to Table 1 the values obtained are very small, although they increase with the presence of NPs. This increase is more significant in the films with PEDOT:PSS, which once again demonstrates better behavior as a matrix as post-treatment with IPA favors the interaction and distribution of NPs in the PEDOT:PSS. Finally, and as expected, it is observed that the deformation in the hybrid films is smaller than in the films with the pristine polymers; the NPs exert the function of anchoring sites that prevent the flow and deformation of the matrix. For applications in organic electronics, it is required that the films have adequate mechanical resistance and dimensional stability, which according to the results obtained is favored by incorporating the NiFe_2_O_4_ NPs in the PEDOT:PSS matrix.

**Figure 6:**
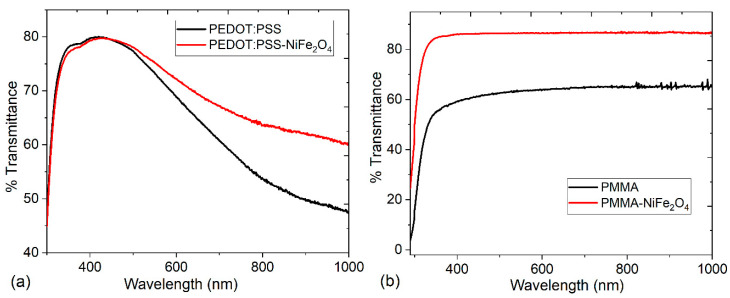
Spectral behavior of the transmittance for (**a**) PEDOT:PSS and (**b**) PMMA pristine and hybrid films.

The optical and electrical behavior of the hybrid films must also be evaluated to determine if they have a potential for use in device manufacturing. In order to evaluate the ability of the films to behave as transparent anodes, the transmittances of the pristine polymers and the hybrid films PEDOT:PSS-NiFe_2_O_4_ NPs and PMMA-NiFe_2_O_4_ NPs were evaluated. The spectra of Figure 6 show that the presence of NiFe_2_O_4_ NPs increases the transmittance in the hybrid films. This effect is more significant in the PMMA-NiFe_2_O_4_ NPs film, where a higher transmittance of 87% is obtained compared to the 80% that is achieved at wavelengths between 340 and 470 nm in the hybrid film with PEDOT:PSS. This result suggests the possibility of using PMMA-NiFe_2_O_4_ NPs film as a transparent anode, which could eventually replace costly or easily oxidized anodes such as ITO or FTO (fluorine-doped tin oxide), which are just some of the expensive anodes currently used. In solar cells, for example, radiation must enter the interior of the device and reach its active layer, so one of its electrodes, which is normally the anode, should be (semi)transparent to radiation. According to these results, the PMMA-NiFe_2_O_4_ NPs film is a viable option and an even better one than PEDOT:PSS, which has already been studied for this particular application [43,44,45].

If the use of hybrid films as constituents of devices in organic electronics is required, it is important to determine their optical band gap (E_g_), which indicates their charge transport capability. The model by Tauc [46,47] relates the E_g_ of hybrid films to its photon energy (*hν*) and the absorption coefficient (*α*) according to:

(*αhν*) = A(*hν* − E_g_)^r^

In the above expression, A depends on the type of transition, r takes the value 2 for indirect electronic transitions in amorphous heterostructures, *h* is Planck’s constant, and *ν* is the frequency, which is given by:

$$\nu =\frac{c}{\lambda }$$

where *c* is the speed of light, *λ* is the wavelength, and *α* is experimentally obtained from [47]:

*α* = (1/*d*) ln (1/*T*)

**Figure 7:**
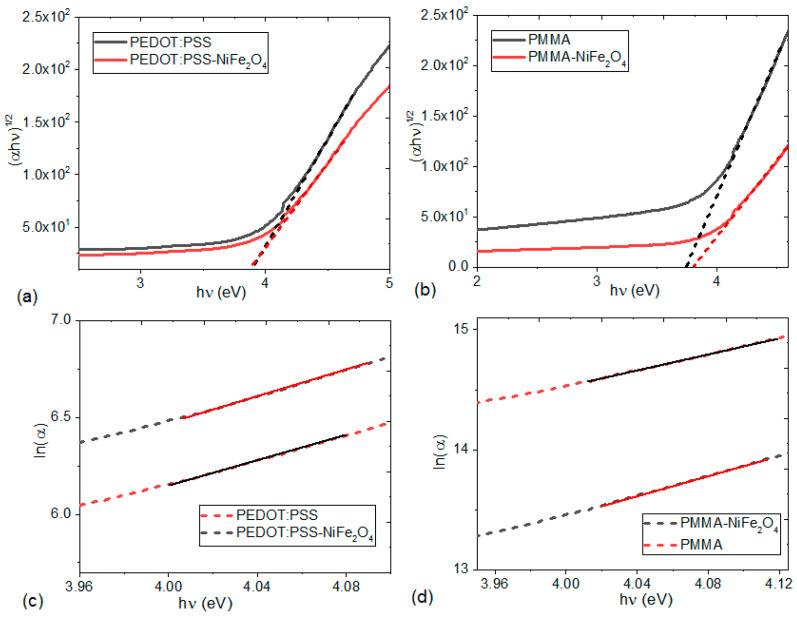
Variation of (*αhν*)^1/2^ with *hν* for hybrid films of (**a**) PEDOT:PSS and (**b**) PMMA. Variation of ln(*α*) with *hν* for hybrid films of (**c**) PEDOT:PSS and (**d**) PMMA.

Here, *d* is the film thickness as obtained from AFM and *T* is the transmission; the reflectivity (R) is neglected due to its low level for each film, which is caused by the presence of NPs [33,48]. The Tauc band gap is determined from the graphs of Figure 7 by plotting (*αhν*)^1/2^ versus *hν* and finding the intercept on the *hν* axis through extrapolation of the plot to (*αhν*)^1/2^ = 0. In these graphs, a similarity is observed when comparing the curves for the films of the pure PEDOT:PSS and PMMA polymers and those for their films with NiFe_2_O_4_ NPs. The Tauc bandgap value reported in Table 2 is the same for PEDOT:PSS pristine and for the PEDOT:PSS-NiFe_2_O_4_ NPs film. According to Jarząbek et al. [48], this constant value of the Tauc band gap means that the NPs do not influence conjugation in the main, rigid polymer chains. It seems that the structural changes of the composite film during the incorporation of the NPs and the post-treatment with IPA are due to the redistribution of NPs in the polymeric matrix, but the conjugation in the main PEDOT:PSS chains is preserved and the energy band gap remains constant. Regarding the band gap of the PMMA and PMMA-NiFe_2_O_4_ films, the introduction of the NPs influences the conjugation in the main chains of PMMA. The films with PMMA have the lowest Tauc gap and would therefore have a behavior resembling that of a semiconductor. The Tauc gap is a consequence of several factors related to electronic-transition processes, including defects, structural disorder, and traps. The Urbach energy (*E_U_*) can be used to determine the defects in the band gap and can be evaluated from the following equation [49,50]:

$$\alpha =A_{a}exp\left(\frac{hv}{E_{U}}\right)$$

In addition to the parameters defined above, $A_{a}$ is a constant of the material that conforms to *α* at the energy gap. Figure 7c,d displays the nearly linear relationship between ln(*α*) and *hν* for the hybrid films. The values of *E_U_* were determined from the reciprocal of the slope from this linear relation and have been recorded in Table 2. The Urbach energy corresponds to the width of the band tail, which is related to localized states within the energy gap, possibly caused by structural defects [48]. As a reference, the value of *E_U_* is zero in a perfect semiconductor [51]. The highest *E_U_* belongs to the PEDOT:PSS-NiFe_2_O_4_ NPs film, and the lowest *E_U_* belongs to the PMMA-NiFe_2_O_4_ NPs film. From the values of E_g_ and *E_U_*, it is understood that the PMMA film shows the best semiconducting behavior, which is a required feature in organic electronics. In addition to performing as a transparent anode (see Figure 6b), it also has the potential for use as an active layer. The behavior of the NPs and their effect on the optical properties of the films depend on the matrix in which they are embedded. In the case of films with PEDOT:PSS, the presence of NPs slightly increases the value of *E_U_*; however, the value of *E_U_* for the PMMA pristine matrix is higher than that obtained with the NiFe_2_O_4_ NPs. Finally, it is important to consider that the values obtained for *E_U_* are in the same range as those for some inorganic semiconductor films, such as Bi_2_S_3_ (0.26 eV) and Bi_2−x_Cr_x_S_3_ (0.35 eV) [51]. They are lower than for some hybrid films, such as PVOH (polyvinyl alcohol) film embedded with Bi-nanoparticles (0.604 eV) [52], while they are bigger than for hybrid films with poly(3-hexylthiophene) (0.11–0.17 eV) [48]. Unfortunately, there is not much information available about the *E_U_* values for hybrid films with FeNi_2_O_4_ NPs in a polymeric matrix.

**Figure 8:**
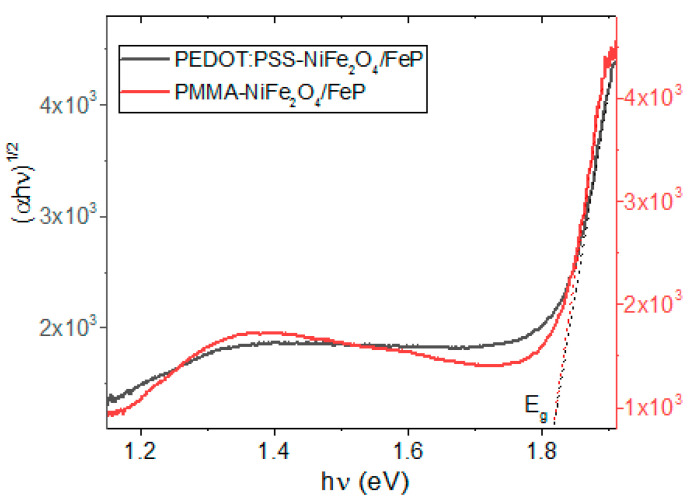
Variation of (*αhν*)^1/2^ with *hν* for hybrid films with a PFeCl layer.

To improve the semiconducting behavior of the hybrid films, a PFeCl layer was deposited by evaporation under high vacuum, forming a planar heterojunction on the polymer-NiFe_2_O_4_ NPs films. The porphine film was added as an electron transport layer. According to Figure 8, the presence of PFeCl film with a low band gap could favor a semiconductor behavior in the/polymer-NiFe_2_O_4_ NPs/PFeCl/system. The narrow band gap can be attributed to (i) FePCl optical properties, (ii) their proper interaction with the components of the hetero-junction system [23], and (iii) heat-induced movements of polymer particles [48,53]. During the deposition of the PFeCl film by the high-vacuum evaporation technique, the porphine molecules in a gaseous state are deposited on the polymer-NiFe_2_O_4_ NPs film, and at this site they turn to the solid state. While this deposition occurs, the polymer-NiFe_2_O_4_ NPs film heats up, thus enlarging the free volume and decreasing the order between polymer chains, which causes a blue shift and a decrease in the band gap [48,53].

**Figure 9:**
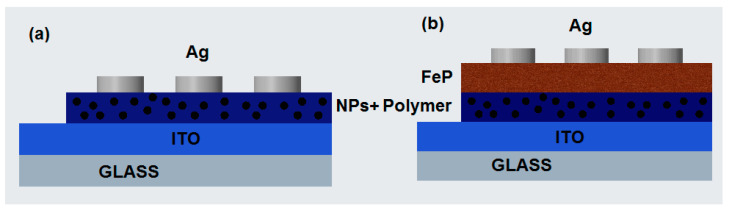
Device scheme for (**a**) glass/ITO/polymer-NiFe_2_O_4_ NPs/Ag and (**b**) glass/ITO/polymer-NiFe_2_O_4_ NPs/PFeCl/Ag devices.

**Figure 10:**
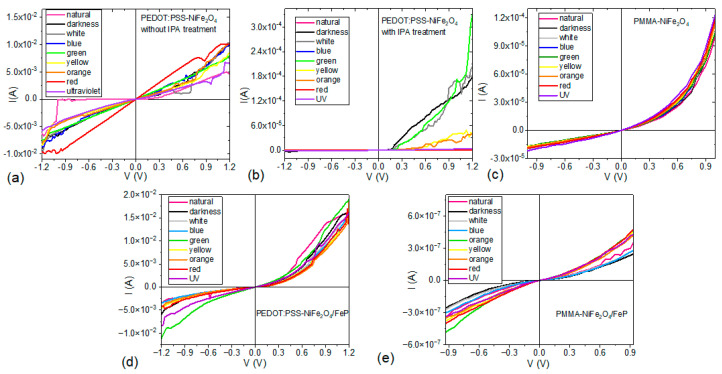
I-V curves from (**a**–**c**) glass/ITO/polymer-NiFe_2_O_4_ NPs/Ag and (**d**,**e**) glass/ITO/polymer-NiFe_2_O_4_ NPs/PFeCl/Ag devices under different lighting conditions.

To evaluate the electrical behavior in the heterostructures, Figure 9 shows the schemes of the devices manufactured for the polymer-NiFe_2_O_4_ NPs (Figure 9a) and/polymer-NiFe_2_O_4_ NPs/PFeCl/(Figure 9b) devices. Figure 10a,b shows the behavior of the devices with PEDOT:PSS before and after performing the IPA treatment. When comparing the graphs, a marked difference between the two is observed as a result of the rearrangement between the NPs and the polymer after post-treatment. Before the treatment, an ohmic behavior is observed in the device under the different lighting conditions; in addition, there is a tendency towards the ambipolarity of the device. However, under most lighting conditions and at V > 0.4 V, the device interferes with the current flow, probably because the NPs are not perfectly embedded in the polymeric matrix. After treatment, the behavior of this device changes completely; according to Raman spectroscopy, the post-treatment with IPA changes the distribution of NPs in the polymeric matrix, and this influences charge transport. When analyzing and comparing the results obtained for the glass/ITO/polymer-NiFe_2_O_4_ NPs/Ag devices with PEDOT:PSS (see Figure 10b) and PMMA (see Figure 10c), it is observed that the maximum current carried is of the same order of magnitude in the two devices; the device with PEDOT:PSS is affected by the type of radiation that falls on it. The maximum current carried is generated with wavelength illumination corresponding to green light, while a current reduction occurs under blue and violet lighting or under natural lighting conditions. The behavior of this device in darkness also changes, and this result could be an indication of photovoltaic properties that may be studied in the future. The type and position of the anode and cathode, as well as the direction of current flow, determine device behavior; at negative voltages, the behavior is practically that of an insulator, while at positive voltages, and despite presenting interference in charge transport, the behavior is ohmic. On the other hand, in the device with PMMA, there is no significant influence on charge transport when lighting conditions change; the behavior is mostly ohmic, although it is somewhat similar to that of a Schottky diode. In this device, there is no ambipolar behavior; nevertheless, current flow is continuous and without barriers or interference generated by the NPs-polymer interface.

The incorporation of the porphine film generates a significant change in the electrical behavior of the devices, which can be considered ambipolar (see Figure 10c,d). Transported current increases by two orders of magnitude in glass/ITO/PEDOT:PSS-NiFe_2_O_4_ NPs/Ag devices relative to glass/ITO/PEDOT:PSS-NiFe_2_O_4_ NPs/PFeCl/Ag devices. On the other hand, through the incorporation of the porphine film, the electric current decreases by three orders of magnitude in the glass/ITO/PMMA-NiFe_2_O_4_ NPs/Ag/device relative to the glass/ITO/PMMA-NiFe_2_O_4_ NPs/PFeCl/Ag/device. The devices with the PFeCl film show a behavior resembling that of a Schottky diode, and there are no significant changes when irradiating them with different wavelengths. Finally, it is important to mention that the device with the highest current is glass/ITO/PEDOT:PSS-NiFe_2_O_4_ NPs/PFeCl/Ag/. From these results, it can be seen that the rectifying behavior of the devices without the porphine film acquires an ohmic character with the addition of PFeCl. In the PEDOT device, this also leads to an increased electric current, whereas the PMMA device undergoes a reduction in current, which can be attributed to changes in the local electronic structure at the interface between the polymer and the porphine films.

**Figure 11:**
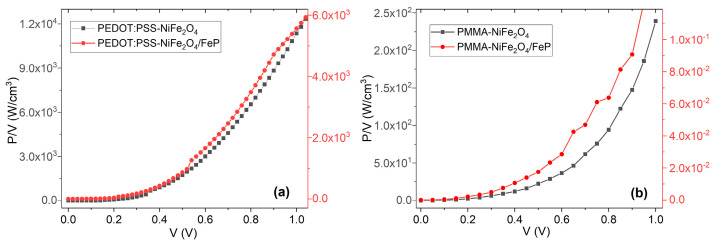
P-V curves from (**a**) PEDOT:PSS and (**b**) PMMA devices under natural lighting conditions.

Figure 11 shows the graphs of power per unit volume with respect to (positive) voltage applied to the devices. Device volumes are 3.78 × 10^−7^ and 2.62 × 10^−6^ cm^3^ for the glass/ITO/PEDOT:PSS-NiFe_2_O_4_ NPs/Ag and glass/ITO/PEDOT:PSS-NiFe_2_O_4_ NPs/PFeCl/Ag devices, respectively, and 4.21 × 10^−7^ and 2.98 × 10^−6^ cm^3^ for the glass/ITO/PMMA-NiFe_2_O_4_ NPs/Ag and glass/ITO/PMMA-NiFe_2_O_4_ NPs/PFeCl/Ag devices, respectively. As expected, there is a roughly exponential shape in the power density versus voltage graphs that can be associated to the rectifier- and ohmic-like behaviors of the devices. It is interesting to notice the discontinuous changes in slope in the graph of the PMMA-based device, which are possibly due to charge-storage effects at the interfaces between the nanoparticles and the matrix.

## 4. Conclusions

Hybrid films of NiFe_2_O_4_ NPs in PEDOT:PSS and PMMA were obtained by the spin-coating technique. The NPs positively affect the films’ mechanical properties by increasing the maximum stress they can withstand and their HK hardness. The behavior of the NPs and their effect on the optical properties of the films depend on the matrix in which they are embedded. Another important aspect of the use of NPs is their effect on the electrical behavior of glass/ITO/polymer-NiFe_2_O_4_ NPs/Ag devices, which changes depending on the matrix. In the case of PEDOT:PSS, IPA treatment influences charge transport, and the current carried in its device depends on the wavelength with which it is irradiated. In the case of the device with the PMMA matrix, an ambipolar behavior occurs which is independent of the light that falls on it. NiFe_2_O_4_ NPs may be considered for use in organic electronics applications; when used as film reinforcement with PEDOT:PSS and PMMA matrices, they enhance their mechanical, optical, and electrical properties. The addition of a porphine layer to devices based on these materials adds an ohmic component to their mostly rectifying behavior.

## Figures and Tables

**Table 1:** Roughness and mechanical parameters of polymeric and hybrid films.

Film	RMS (nm)	Ra (nm)	σ_max_ (MPa)	HK_max_	ε_unit_
PEDOT:PSS	2.97	2.27	10.8	0.039	0.95
PEDOT:PSS-NiFe_2_O_4_ NPs	19.93	15.01	28	0.103	0.92
PMMA	3.09	2.24	8.06	0.029	0.95
PMMA-NiFe_2_O_4_ NPs	18.65	13.32	8.2	0.030	0.91

**Table 2:** Optical band gap and Urbach energy of PEDOT:PSS and PMMA hybrid films.

Sample	E_g_ (eV)	E_U_ (eV)
PEDOT:PSS	3.89	0.318
PEDOT:PSS-NiFe_2_O_4_ NPs	3.89	0.331
PMMA	3.75	0.298
PMMA-NiFe_2_O_4_ NPs	3.81	0.244

## Data Availability

Data is contained within the article.
